# Analysis of the Effect of Antibiotic Bone Cement in the Treatment of Diabetic Foot Ulcer through Tibia Transverse Transport

**DOI:** 10.1111/os.13412

**Published:** 2022-08-05

**Authors:** Xiaofang Ding, Yusong Yuan, Hao Lu, Yuanli Wang, Kunyu Ji, Haorun Lv, Hailin Xu, Junlin Zhou

**Affiliations:** ^1^ Department of Orthopaedics, Beijing Chaoyang Hospital Capital Medical University Beijing China; ^2^ Department of Orthopaedics Beijing Longfu Hospital Beijing China; ^3^ Orthopaedic Trauma Department China‐Japan Friendship Hospital Beijing China; ^4^ Department of Trauma and Orthopaedics Peking University People's Hospital, Peking University Beijing China; ^5^ Key Laboratory of Trauma and Neural Regeneration (Peking University) Ministry of Education Beijing China; ^6^ Diabetic Foot Treatment Centre Peking University People's Hospital, Peking University Beijing China; ^7^ Department of Orthopaedics National Center for Trauma Medicine Beijing China

**Keywords:** Antibiotic bone cement, Diabetic foot ulcer, Infection, Tibial transverse translation

## Abstract

**Objective:**

To explore the efficacy of antibiotic bone cement (ABC) combined with the modified tibial transverse transport (mTTT) on the treatment of severe diabetic foot with infection.

**Methods:**

A retrospective cohort study was conducted of 243 patients with TEXAS grade 3/4 stage D diabetic foot ulcers from December 2016 to December 2019. A total of 115 patients treated with mTTT were classified as the mTTT group (78 male and 37 female, mean age: 70.4 ± 6 years) and 128 patients who were treated with ABC combined with mTTT were in the ABC + mTTT group (89 male and 39 female, mean age: 68.9 ± 8 years). Follow‐up records during treatment and 6 months after surgery were collected, including the time required for white blood cells (WBC) and C‐reactive protein (CRP) to return to normal range, wound healing time, pain visual analog scale (VAS), ankle‐brachial index (ABI), foot skin temperature, transcutaneous oxygen pressure measurement (TcPO_2_), complications, and other indicators. Normally distributed data were compared using the independent sample t‐test, non‐normally distributed data were analyzed by one‐way ANOVA analysis of variance.

**Results:**

There were 128 cases in the ABC + mTTT group (89 male and 39 female, mean age: 68.9 ± 8 years) treated with ABC and mTTT, and 115 cases in the TTT group (78 male and 37 female, mean age: 70.4 ± 6 years) treated with mTTT alone. The time required for WBC and CRP to return to the normal range and wound healing time in the ABC + mTTT group were significantly shorter than those in the mTTT group (12.9 ± 4.6 *vs.* 22.6 ± 1.6 days, *t* = 3.979, *p* < 0.001; 25.3 ± 1.3 *vs.* 31.3 ± 2.3 days, *t* = 4.261, *p* = 0.001; 11.9 ± 3.8 *vs.* 15.9 ± 3.9 days, *t* = 4.539, *p* < 0.001). There were no significant intergroup differences in the foot skin temperature, VAS score, ABI, and TcPO_2_ (*t* = 0.349, 0.542, 0.765, 0.693 while all *p* > 0.05).

**Conclusion:**

Although the application of ABC with mTTT for treatment of diabetic foot ulcers did not affect the wound healing time and ankle blood supply in the mid‐term, it could control ulcer infection faster and accelerate wound healing.

## Graphical abstract

The antibiotic bone cement (ABC) was shaped and perforated according to the shape and size of the wound. The cooled ABC was filled in the infection and tissue defects, covering the wound surface, and fixing it on the wound surface with sutures. The auxiliary materials were replaced every day or every other day according to the exudation of the wound. The ABC was retained for 4–8 weeks, and the decision to continue covering with the ABC depended on the culture results of the wound secretion. It was certified that this technique could control ulcer infection faster than traditional methods and accelerate ulcer wound healing.

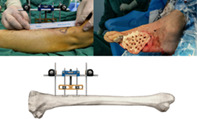

## Introduction

Diabetic foot ulcer refers to foot ulcers in diabetic patients due to neuropathy and various degrees of peripheral vascular disease. The main manifestations are ulcer formation and/or deep tissue destruction with or without foot infections.^1^ The incidence of diabetic foot ulcers is about 8.1% per year, and the recurrence rate of diabetic foot ulcers in the first year is 31.6%.^2^ The treatment of diabetic foot is costly, and the final amputation rate can be as high as 85%; the 5‐year survival rate of patients after amputation is less than 50%.^3^

In view of the different characteristics of diabetic foot ulcers, the TEXAS classification standard^4^ divides diabetic foot ulcers into four grades, and each grade contains four stages that can better reflect the role of blood supply and infection in the development of ulcers. Among them, TEXAS grades 3 and 4 denote the involvement of the ulcer surface and deep tissues, respectively, and D stage indicates ischemia and infection.

As a terminal complication of diabetes, diabetic foot ulcers have complex pathogenesis and are related to multiple medical disciplines. At present, the joint action of microcirculation disorder and neurodegeneration is considered to lead to the occurrence of diabetic foot, wherein foot infection will aggravate the process of ulcer and even gangrene.^5^ Therefore, the establishment of a joint diabetic foot clinic and a multidisciplinary approach to treat diabetic foot ulcers has gradually been accepted.^6^

Tibial transverse transport (TTT) was a new technique for the treatment of diabetic foot ulcers conducted by Chinese doctors through the Stress–Strain Law of Ilizarov. The basic principle of TTT is to generate local tension stimulation through the continuous and slow transfer of the external fixator, combined with the tension‐stress law, thereby promoting the regeneration of local tissues and blood vessels, or causing the recanalization of microcirculatory blood vessels. Pathological studies have confirmed that the proliferation of vascular endothelial cells, the formation of new capillaries and tiny arteries can be observed by this technique, and the formation of thrombus was not observed in these new blood vessels.^7^

In this study, modified tibial transverse transport (mTTT) combined with antibiotic bone cement (ABC) was used to treat TEXAS grades 3/4 and stage D diabetic foot ulcers, and the disease outcome of patients who received the combination therapy was retrospectively analyzed. The retrospective study aimed to (i) compare the advantages and disadvantages of combined ABC and TTT in the treatment of infected diabetic foot and traditional antibacterial methods in the treatment of infected diabetic foot and (ii) evaluate the effectiveness of the combination treatment on severe diabetic foot ulcers.

## Materials and Methods

### Inclusion and Exclusion Criteria

Patients who met the following criteria at the same time were included in the study: (i) diabetic foot who met the diagnostic criteria for diabetic foot proposed by the International Working Group on the Diabetic Foot^8^; (ii) Texas classification of grade 3/4 stage D; (iii) clear mental abilities and without psychiatric diseases, and who could cooperate in the research and treatment; (iv) received ABC treatment or systemic anti‐infective treatment; (v) patients with complete clinical data and follow‐up time >6 months.

Patients who met any of the following criteria were excluded from the study: (i) severe systemic infection and in need of immediate amputation to prevent the development of disease and save lives; (ii) mental illness and who could not cooperate to complete the adjustment and care of the external fixator; (iii) severe cardiovascular and cerebrovascular diseases (such as severe cardiac insufficiency, severe sequelae of cerebrovascular disease) and acute infectious diseases; (iv) receiving other ulcer wound treatment methods; (v) abnormal liver and renal function.

### Patient Demographics

A total of 243 patients with diabetic foot ulcers who were treated at Peking University People's Hospital and Beijing Longfu Hospital from December 2016 to December 2019 were included in this study. In all, 115 patients treated with mTTT were classified as the mTTT group (78 male and 37 female, mean age: 70.4 ± 6 years). Of these, 26 and 89 patients were in the 3D and 4D stage as per the TEXAS classification (Table 1), respectively. Further, 128 patients who were treated with ABC combined with mTTT were in the ABC + mTTT group (89 male and 39 female, mean age: 68.9 ± 8 years). Of these, 32 and 96 cases were in the 3D and 4D stage, per the TEXAS classification (Table 1). This study has been approved by Beijing Longfu Hospital Ethic Committee (No. LFYYLL‐2021‐24).

**TABLE 1:** Demographic of patients

Demographic	MTTT group	Experiment group	*p* value
Gender (n, n%)			0.775
Female	37 (32.17%)	39 (30.47%)	
Male	78 (67.83%)	89 (69.53%)	
Age (years)	70.4 ± 6	68.9 ± 8	0.326
Diabetes type (n, n%)			0.914
Type I	2 (1.74%)	2 (1.56%)	
Type II	113 (98.26%)	126 (98.44%)	
DF course (months)	3.8 ± 0.4	4.3 ± 0.1	0.157
TEXAS classification (n, n%)			0.662
3D	26 (22.61%)	32 (25%)	
4D	89 (77.39%)	96 (75%)	
Pre‐op WBC (×10^9^/L)	16.53 ± 3.78	15.68 ± 4.54	0.812
Pre‐op CRP (mg/L)	168 ± 5.43	148 ± 6.4	0.718
Instep epidermis temperature (°C)	30.3 ± 0.69	30.5 ± 0.98	0.956
VAS	8.2 ± 0.71	7.9 ± 0.86	0.876
ABI	0.29 ± 0.30	0.32 ± 0.31	0.427
TcPO_2_ (mmHg)	30.1 ± 1.6	29.4 ± 1.34	0.863

Abbreviations: ABI, ankle brachial index; CRP, C reactive protein; DF, diabetic foot; Pre‐op, pre‐operation; TcPO2, transcutaneous (partial) pressure of oxygen; VAS, visual analog scale; WBC, white blood cell.

### General Treatments

At the start of the treatment, both endocrinologists and vascular surgeons were consulted to monitor the patient's blood sugar and evaluate the vascular condition of their limbs, adjust blood glucose fluctuations (target blood glucose control standards: rapid blood glucose before meals <7 mmol/L and rapid blood glucose 2 h after meal <11 mmol/L). All exudates from foot ulcers underwent bacterial culture and drug susceptibility testing; these results were the basis for selection of sensitive antibiotics for intravenous administration. Emergency debridement, incision and drainage, and foot care was performed on all patients with foot necrotic tissue.

### Surgery

#### mTTT

The detailed surgical procedure can be referred to in a previous article.^9^ Two 3.0 Steinmann pins were inserted through the single layer of cortical bone. The Steinmann pins were used as the centre point for the drilling on four sides with a 2.0 drill bit and use of a rapid osteotomy device; the length of each side was 1.5 cm. Subperiosteal osteotomy was performed with a 5‐mm narrow bone knife at an angle of 15°–30° to the bone surface. The external fixators were fixed with 4.0 Steinmann pins at the distal and proximal ends. The subcutaneous tissue and skin were sutured.

#### ABC Implantation

**Fig. 1:**
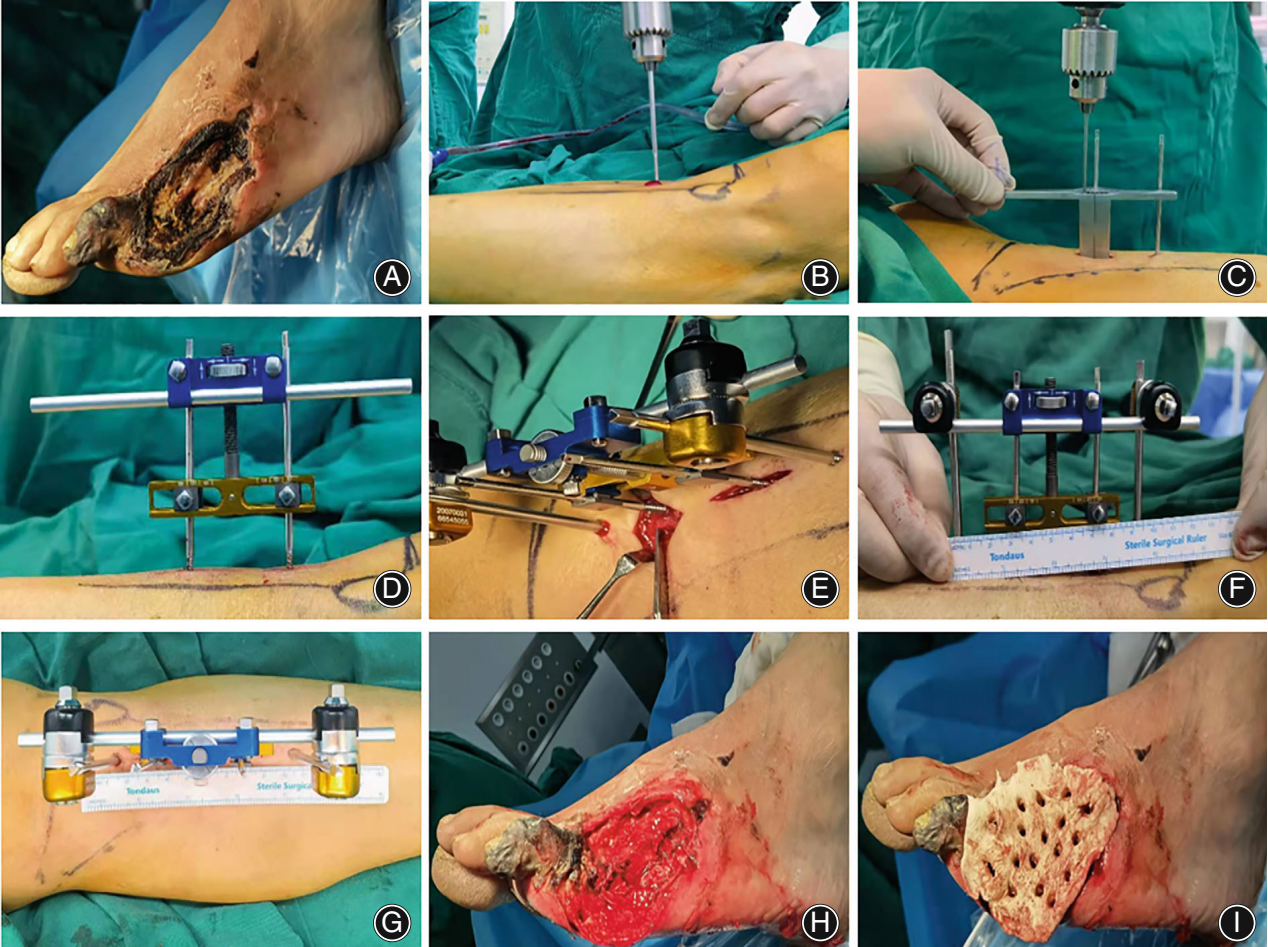
Surgical flowchart. (A) Preoperative ulcer surface: (B) Traction guide needle positioning; (C) Osteotomy perforation; (D) Installation of the connecting rod and traction needle knob with the traction needle as the centre; (E) Transverse subperiosteal osteotomy was performed after installing the fixed needle; (F, G) Assembling the remaining part of the external fixator; (H, I) After debridement, the wound is implanted with antibiotic bone cement

Conventional 40 g gentamicin bone cement (Depuy Synthes, Johnson & Johnson) was used as the matrix to mix the sensitive antibiotics to make the ABC needed for treatment after debridement of the ulcer surface (vancomycin was reserved as a special medicine to gram‐positive bacteria, and ceftazidime was used to gram‐negative bacteria). The ABC was shaped and perforated according to the shape and size of the wound. The cooled ABC was filled in the infection and tissue defects, covering the wound surface, and fixing it on the wound surface with sutures. The auxiliary materials were replaced every day or every other day according to the exudation of the wound. The ABC was retained for 4–8 weeks, and the decision to continue covering with the ABC depended on the culture results of the wound secretion (Figure 1).

#### Postoperative Management

The handling and processing procedures of the tibial bone flap after surgery has been described in a previous study.^8^ Dressing was done according to the wound exudation. The nail passageway was infiltration with 2 mL 75% ethanol three times a day. Postoperative antibiotics were routinely used to control infections, and comprehensive treatment was administered, including standardized insulin therapy, blood sugar control, nutritional nerve drugs to improve nerve tissue growth, correct electrolyte disturbances to maintain homeostasis, oral anticoagulant drugs, oral or intravenous nutritional preparations to regulate protein levels, and homeostasis maintenance by correcting electrolyte imbalance.

#### Follow‐Up

Follow‐up was performed before and 6 months after the operation. Telephone follow‐up and invitation to patient outpatient follow‐up were taken if the patient has been discharged. Observation and evaluation indicators included visual analog scale (VAS); ABI (Vista AVS Peripheral Vascular Diagnostic System); and TcPO_2_ (Danish transcutaneous oxygen partial pressure monitor, TCM400); skin temperature of the foot and ankle (by considering the midpoint of the inner and outer ankle joint line, BOSCH handheld infrared thermometer, GIS500); and TEXAS classification.

#### Statistical Analysis

SPSS (version 11.0, IBM Corporation, Armonk, NY, USA) was used for statistical analysis of the data. Normally distributed data were compared using the independent sample *t*‐test; non‐normally distributed data were analyzed by one‐way ANOVA analysis of variance. Rank data were used to compare the differences between the two groups using the rank sum test. Categorical variables used the chi‐square test to analyze the difference between the two groups. Inspection level α is 0.05.

## Results

### Outcome of the Infection Factors

**Fig. 2:**
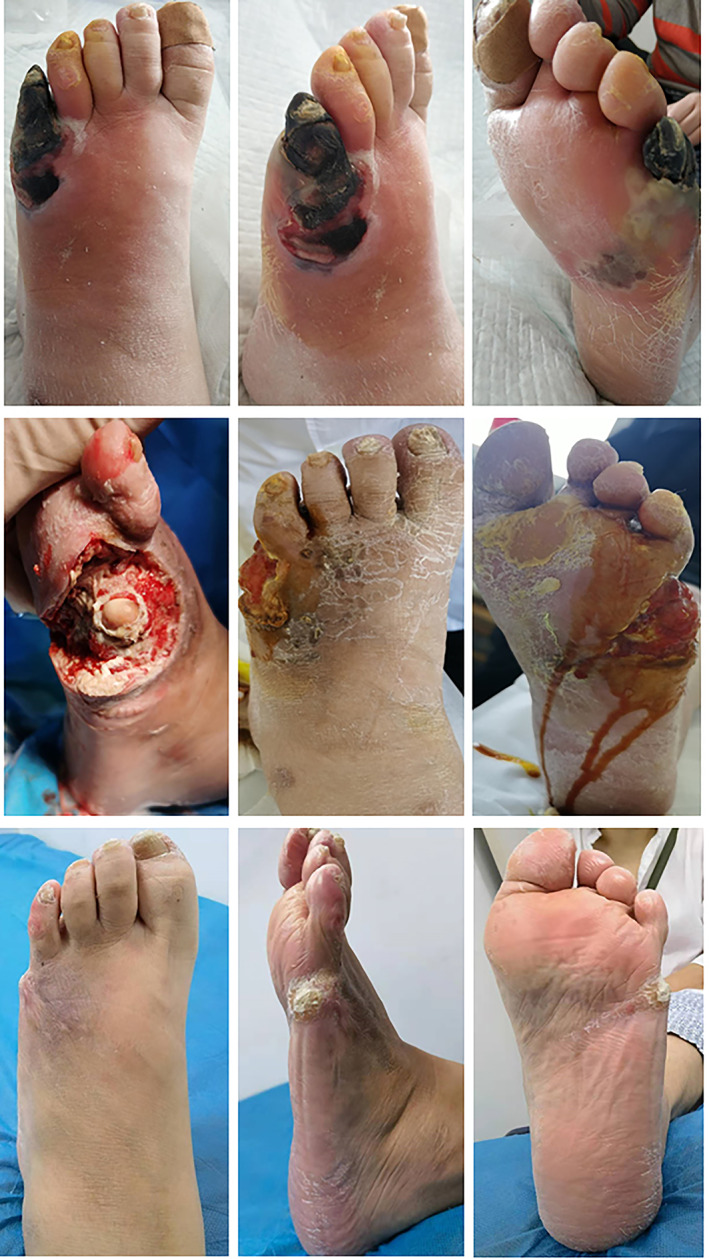
Case representation of the mTTT group. A 43‐year‐old woman with a 10‐year history of diabetes suffered from a diabetic foot ulcer on the fifth toe of her left foot for 6 weeks. The wound was ulcerated and infected. Conservative treatment was ineffective after dressing change and debridement. Admission examination revealed an infectious ulcer of the fifth toe of the left foot accompanied by gangrene. The abscess cavity spread to the fifth metatarsophalangeal joint, accompanied by progressive necrosis of the distal fifth phalanx. The secretions from the wound were thick and foul‐smelling, and the cortical bone was necrotic and had sloughed off. (A–C) The appearance of the ulcer surface before operation; (D) The appearance of the wound after debridement and amputation; (E, F) The appearance of the wound after debridement and dressing change combined with VAC drainage for 6 weeks; (G, H): The wound was completely healed at 19 weeks postoperatively

**Fig. 3:**
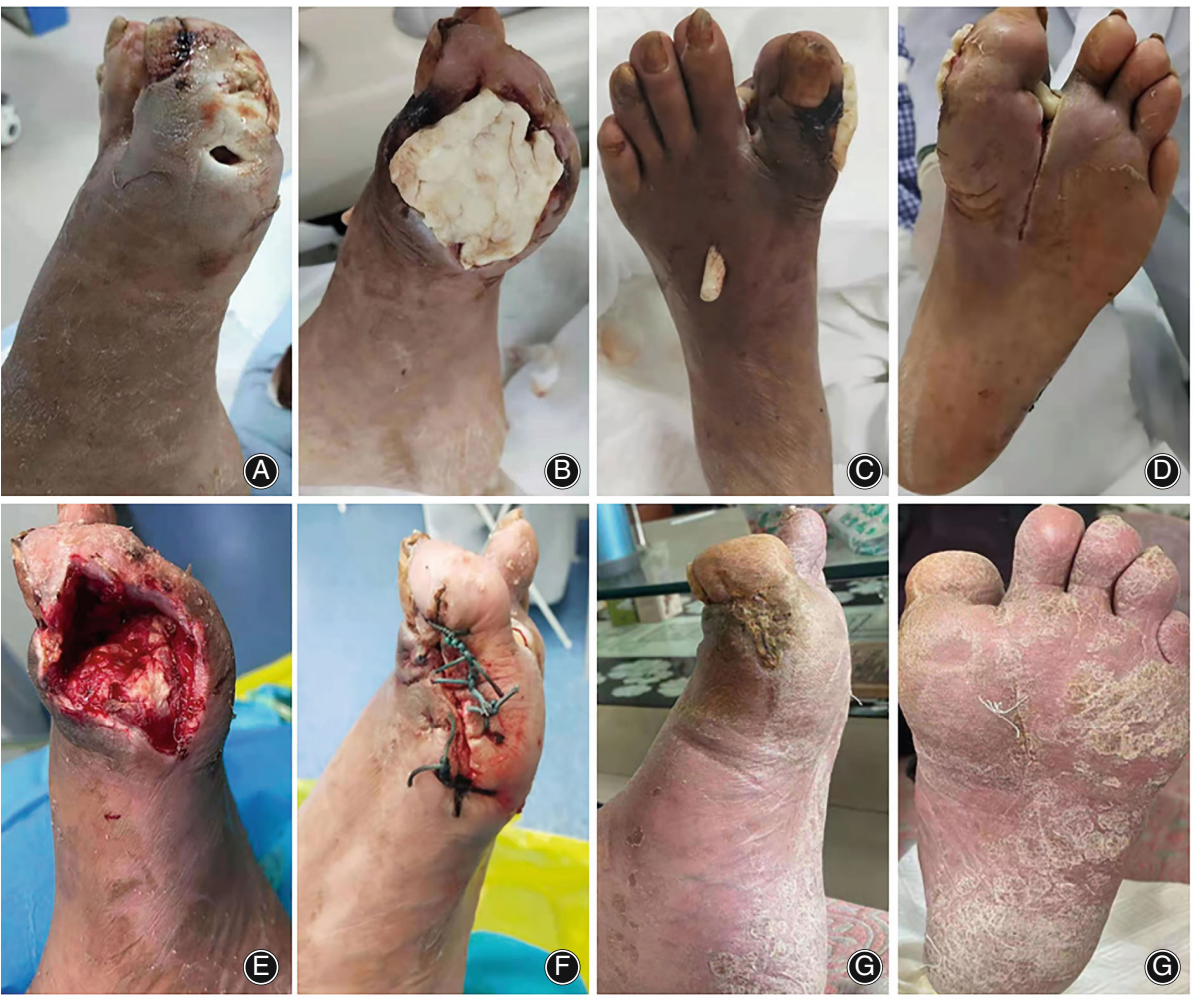
Case representation of the ABC + mTTT group. A 68‐year‐old man with a history of diabetes for 12 years. He suffered from foot ulcers, increased infections, and tissue necrosis for 4 months. Regular dressing changes, debridement, and conservative treatment were ineffective. Admission examination revealed an infectious ulcer between the first metatarsal and the first and second metatarsals of the foot. The abscess cavity had spread to the first metatarsophalangeal joint, accompanied by progressive necrosis of the distal end of the first metatarsal bone. The secretion from the ulcer surface was thick and foul‐smelling, and cortical bone was necrotic and had sloughed off. (A) Lateral view of the ulcer; (B–D) Foot view after implantation of bone cement; (E) Wound condition after removal of bone cement at 6 weeks postoperatively; (F) Closed wound at 8 weeks postoperatively; (G, H) At 13 weeks postoperatively, the wound was completely healed

The follow‐up time was 6–18 (12.6 ± 2.8) months. In the mTTT group, the time required for WBC and CRP to return to the normal range was 22.6 ± 1.6 days and 31.3 ± 2.3 days, respectively, which were significant longer than that of ABC + mTTTgroup (*t* = 3.797, *p* < 0.001; *t* = 4.261, *p* = 0.001) (Figures 2 and 3).

### Outcome of the Healing Factors

Similarly, compared with the mTTT group, the mean healing time of ABC + mTTT group was much shorter (15.9 ± 3.9 *vs* 11.9 ± 3.8 days, *t* = 4.539, *p* < 0.001). There were no significant differences of the skin temperature (35.3 ± 1.24 *vs* 34.8 ± 1.35°C, *p* = 0.126), VAS score (1.1 ± 0.48 *vs* 1.2 ± 0.67, *p* = 0.523), ABI (0.72 ± 0.21 *vs* 0.77 ± 0.35, *p* = 0.267), and TcPO_2_(40.1 ± 3.39 *vs* 41.2 ± 2.78 mmHg, *p* = 0.192) of the affected foot between the two groups.(Figures 2 and 3).

### Classic Case

**Fig. 4:**
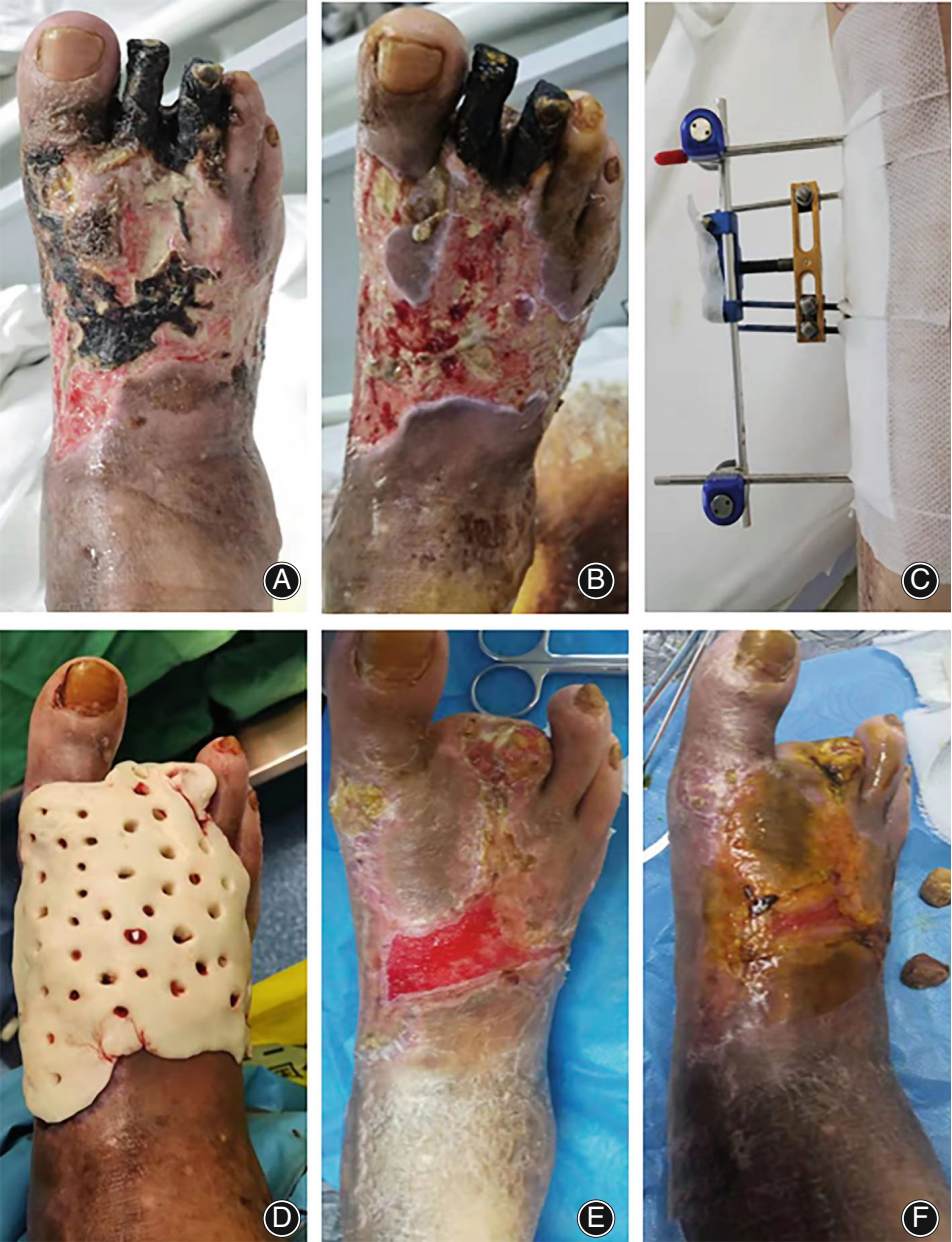
Classic case of ABC + mTTT. (A–F) are images of the patient's wounds at admission, after debridement, mTTT treatment, ABC treatment, at discharge, and at healing, respectively

An elderly male patient was admitted to our hospital for 2 years of recurrent infection in the right foot. He had a history of diabetes. On admission, his fasting blood glucose was 22.2 mmol/L and his white blood cell was 15.5×10^9^/L. Physical examination revealed localized gangrene on the dorsum of the foot, toes, and around the ankle, with severe infection and necrosis. Imaging findings suggested that there was no osteomyelitis. On the fourth day of admission, debridement, amputation, and mTTT were performed. ABC was placed on day 12 of admission. The patient was discharged at 37 days of admission with regular dressing changes. The wound healed on postoperative day 64 without complications (Figure 4).

## Discussion

It was found that the ABC combined with mTTT could control wound infection faster than mTTT and accelerate wound recovery in this study.

### Key Points of Diabetic Foot Ulcer Treatments

Diabetic foot is defined as foot infections, ulcers, and/or deep tissue destruction associated with abnormalities of the distal nerves of the lower extremities and varying degrees of peripheral vascular disease.^4^ The occurrence and development of diabetic foot ulcers are related to neurodegeneration, peripheral blood vessel damage, and infection. Neurodegeneration is generally considered the initiating factor of diabetic foot ulcers. The destruction of microcirculation prolongs the ulceration and makes them difficult to treat and heal, and infections can accelerate the progression of ulcers and aggravate the condition.^10^ The treatment of diabetic foot often begins from these three aspects.

As a terminal complication of diabetes, diabetic foot ulcers cannot be simply regarded as local gangrenous ulcers, rather are a part of the overall disease spectrum. Its pathogenesis is based on a series of pathological changes caused by abnormal glucose metabolism. Diabetes Control and Complications Trial (DCCT), Epidemiology of Diabetes Interventions and Complications (EDIC),^11^ and the United Kingdom Prospective Diabetes Study (UKPDS)^12^ and other large, long‐term follow‐up sample cohorts of type 1 and type 2 diabetes patients have indicated that the application of intensive treatment to nearly normalize blood sugar could reduce the occurrence of microvascular disease. Therefore, blood sugar control is mandatory in diabetic foot ulcer treatment.

### Tibial Transverse Transport

Tibial transverse transport is a new method for the treatment of diabetic foot ulcers based on the Ilizarov stress tension law that has recently been proposed. Many retrospective studies have reported that the effective rate of TTT in the treatment of diabetic foot ulcers was >90%.^8^, ^13^, ^14^, ^15^ Research has shown that after treatment, the proliferation of vascular endothelial cells, formation of new capillaries and arterioles could be observed around the bone flap, and collateral circulation was formed at the end of the lower limbs.^7^ However, it is difficult to completely cure diabetic foot by TTT alone and requires the assistance of other treatment methods.

### ABC for the Infective Wound Curing

Antibiotic bone cement has been used in the treatment of bone marrow infections for a long time and has gradually been included in the treatment system for lower extremity ulcers in recent years. The addition of antibiotics to cement makes the cement both a controlled drug‐release system and a structural stabilizer. Compared with systemic drugs, ABC can maintain a concentration of antibiotics higher than the minimum inhibitory concentration in the lesion, and effectively eliminate pathogenic bacteria in the lesion and its surroundings. Additionally, the drugs released from the local area rarely enter the systemic blood circulation system which reduces the side effects of antibiotics.^16^ Published clinical studies have shown that antibiotic‐loaded bone cement (ALBC) gaskets release sufficient amounts of antibiotics to curb or eradicate poor vascularization and other raging infections in other vulnerable local areas.^17^, ^18^

Vancomycin is a commonly used additive for ABC, and the released local vancomycin concentration is about 0.5–2.0 μg/mL, which can meet the minimum inhibitory concentration requirements.^19^ When evaluating the response breadth of dynamic vancomycin release, studies have found that the plateau period of vancomycin release can be >10 days.^20^, ^21^ New ABCs could be replaced as needed during our treatment, and the drug release curve could meet the treatment needs of diabetic foot ulcers.

The wound secretion culture of patients in the ABC + mTTT group showed that *Staphylococcus aureus* was the most dominant organism and was resistant to many conventional antibiotics. The sensitivity test to vancomycin was finally confirmed. Bone cement was used as the drug delivery carrier and vancomycin was incorporated into bone cement slice for local covering application, which plays the role of slow release of antibiotics, maintains a high concentration of local antibiotics in the wound during a period of wound healing, and induces the production of wound biofilm^22^ that is beneficial to wound repair. Vancomycin has a wide spectrum of sensitive bacteria and can kill most common pathogenic microorganisms.^23^ In the ABC + mTTT group, the infection was effectively controlled after the wound was covered with vancomycin with bone cement as the carrier. Finally, the blood supply was improved by TTT, and the wound was completely healed.

### Innovation of mTTT

In this study, the traditional large arc‐shaped incision for transversal tibial bone transportation with a single long bone flap was modified.^8^ A double linear incision parallel to the long axis of the tibia was adopted to cut the double bone flap for transportation. The incision was smaller than the traditional long arc‐shaped incision. The area was smaller than the 1.5 cm × 5 cm bone window used in some studies, which reduced the occurrence of iatrogenic trauma and incision complications leading to improved healing of calf wounds.

### Advantages of ABC Combined with mTTT

There were no significant intergroup differences with respect to foot skin temperature, VAS score, ABI, and TcPO2 at 6 months after surgery, indicating that regardless of whether combined with ABC for local wound treatment, TTT surgery could effectively restore the microcirculation of the foot and ankle. However, the wound healing time of the ABC + mTTT group was shorter than that of the mTTT group, and the infection index returned to normal significantly earlier than that of the mTTT group, indicating that the combined use of ABC could control infection faster and promote the healing of ulcer wounds.

### Limitations

Our study has some limitations. Some subjective indicators have a degree of recording bias due to the retrospective nature of the study, but objective indicators such as ulcer healing time, complications, and ulcer recurrence rate were accurately recorded that would not affect our overall study conclusions. The strength of this study is that despite being a single‐centre study with certain admission bias, it can provide a reference and research basis for the promotion and further research of this treatment plan in the future.

### Conclusion

In summary, mTTT combined with ABC was a good treatment technique for severe infected diabetic foot ulcers. MTTT combined with ABC could effectively treat TEXAS 3/4 D stage diabetic foot ulcers to control infection faster and achieve better clinical results than mTTT treatment alone.

## Authors' Contributions

Hao Lu and Yuanli Wang were involved in collection and preparation of samples. Kunyu Ji and Haorun Lv did sample analysis. LDC and JRBB. Yusong Yuan and Xiaofang Ding conducted data analysis and wrote the manuscript. Hailin Xu and Junlin Zhou designed this study and edited the manuscript. All authors reviewed the manuscript before submission.

## Ethics Approval

The ethics committee of our institution has approved this study (LFYYLL‐2021‐24).

## Conflict of Interest Statement

The authors have no conflict of interest to disclose.
